# Nitrate contamination characteristics and health risk assessment of groundwater in the typical area of the lower Yellow River Basin

**DOI:** 10.1371/journal.pone.0349752

**Published:** 2026-06-26

**Authors:** Qinghai Deng, Xiao Wu, Wenqiang Zhang, Yue Teng, Changsuo Li, Tianshuo Gu, Caiping Hu, Qingyu Xu, Xiaotian Liu, Shuai Gao

**Affiliations:** 1 Shandong Provincial Geo-mineral Engineering Exploration Institute (801 Institute of Hydrogeology and Engineering Geology, Shandong Provincial Bureau of Geology & Mineral Resources), Jinan, China; 2 College of Earth Science and Engineering, Shandong University of Science and Technology, Qingdao, China; 3 Shandong Engineering Research Center for Environmental Protection and Remediation on Groundwater, Jinan, China; University of Peshawar National Centre of Excellence in Geology, PAKISTAN

## Abstract

Nitrate is a key indicator for assessing groundwater quality, and nitrate pollution in groundwater poses a threat to water security and ecological health in the Yellow River Basin. Taking the Changxiao hydrogeological unit as an example, this paper studies the characteristics, influencing factors and health risks of nitrate pollution in groundwater by using nitrate pollution index, hierarchical cluster analysis, factor analysis, Monte Carlo simulation and human health risk assessment. The results show that the average concentration of NO_3_^-^-N is 19.48 mg/L, with pore water exhibiting the highest levels, followed by karst water and then fissure water in terms of average concentration. The average nitrate pollution index is 3.31, and 50% of the samples are severely polluted with nitrate. The pollution is mainly distributed in densely populated areas, especially near Xiaoli Town, Guide Town and Ancheng Town. Both anthropogenic and natural factors affect the groundwater chemical field in the study area. In areas with serious nitrate pollution, the impact of human activities is particularly serious. Probabilistic health risk assessment shows that the probabilities of potential risks posed by nitrate in pore water to children and adults are 24.37% and 6.67%, respectively; and in karst water, the probabilities to children and adults are 24.83% and 3.64%, respectively. The potential risk of nitrate to adults is far less than that to children who requires special attention. According to sensitivity analysis, the concentration of nitrate is the main variable affecting health risk assessment, which should be paid attention to reduce health risks. These results provide a scientific basis for groundwater pollution control and rational water resource management in both the study area and the lower Yellow River reaches.

## 1 Introduction

Groundwater, as the largest available freshwater resource in the world, plays an important role in maintaining ecological balance and regional development [1]. In recent years, with population growth, urban expansion, and industrial and agricultural development, water environment quality has faced severe pressure in China, and water pollution caused by human activities has become increasingly prominent [2]. Nitrate (NO_3_^-^), a major component of dissolved inorganic nitrogen in natural water bodies that is widely present in aquatic environments and characterized by easy migration and relative stability, has become one of the primary groundwater contaminants attracting extensive research attention worldwide [3–5]. Nitrate in groundwater enters the human body primarily through drinking water consumption and dermal contact, and excessive intake may lead to methemoglobinemia, thyroid dysfunction, hypertension, and other serious health consequences [6–8]. Understanding the current situation and influencing factors of nitrate pollution in groundwater is beneficial for formulating reasonable prevention and control measures, protecting the ecological environment and human health.

The current situation of nitrate pollution in groundwater and its health risks are important issues of societal concern today [2,9]. The nitrate pollution index (NPI) is a method to evaluate the nitrate pollution level in groundwater by using the index to measure the nitrate pollution degree [10,11]. High nitrate content not only causes water pollution, but also poses a great threat to human health [12]. The human health risk assessment model proposed by the United States Environmental Protection Agency (USEPA) is widely used [13,14]. However, the human health risk assessment process involves inherent uncertainties and variabilities, where traditional risk assessment model is inevitably affected by the loss of information and uncertainty risk [9]. Monte Carlo simulation provides a probabilistic risk assessment to reduce the uncertainty of risk, which is applied to the risk assessment of a variety of pollutants [15,16].

Multivariate statistical analysis serves as a powerful analytical method for uncovering complex relationships within multi-variable datasets, enabling the elucidation of interactions among different factors or processes that influence groundwater chemistry and its evolution, thereby facilitating the identification of key factors governing nitrate contamination [17,18]. Commonly used multivariate statistical analysis includes hierarchical cluster analysis [19,20], factor analysis [21] and principal component analysis [20,22]. Hierarchical cluster analysis classifies samples according to the similarity, and elucidates the main influencing factors for each sample group. Factor analysis describes the potential relationship among many variables and identifies the main factors controlling groundwater chemistry. Combined with hierarchical cluster analysis and factor analysis, the main processes affecting the chemical characteristics of groundwater can be analyzed from the perspective of samples and variables, and the factors affecting nitrate pollution can be revealed.

The Yellow River Basin, endowed with abundant natural resources and location advantages, has driven economic development along its reaches. As an important agricultural production base, intensive agricultural activities have led to increasing threats of nitrogen pollution in groundwater [23,24]. The Changxiao hydrogeological unit is located in the lower reaches of the Yellow River, which is an important water supply source in Jinan, Shandong Province, China, with dense population and frequent human activities. Groundwater serves as a vital water resource for local communities, yet faces challenges of nitrate contamination. In recent years, many scholars have conducted research on groundwater in this area. Yu et al. [25] used hydrochemical data and isotope data to determine the recharge source of Changxiao water source area and the impact of the Yellow River; Zhang et al. [26] studied sources of nitrate pollution using isotope data; Liu et al. [27] revealed the enrichment mechanism of karst groundwater in the Changxiao karst water system. In addition, previous researchers have conducted extensive studies on the hydrochemical and hydrogeological characteristics of local groundwater in this area [28,29]. However, health risks associated with nitrate contamination have not been studied. Therefore, this study comprehensively utilized multiple methods to construct a complete research chain from “pollution status identification” to “cause analysis” and then to “risk assessment,” and adopted probabilistic health risks to more accurately characterize the uncertainty of risks. The main objectives are: (1) to clarify the characteristics of nitrate pollution in groundwater; (2) to evaluate nitrate pollution level; (3) to identify the key factors influencing nitrate pollution; (4) to evaluate the potential risk of nitrate on human health. It provides research ideas and reference for groundwater pollution control in typical areas in the lower reaches of the Yellow River Basin.

## 2 Materials and methods

### 2.1 Study area

The Changxiao hydrogeological unit is located in the lower reaches of the Yellow River Basin, characterized by a temperate semi-humid monsoon climate with four distinct seasons. The annual average temperature is 12–14°C and the annual average precipitation is 657.7 mm (1951–2022). Precipitation is unevenly distributed throughout the year, mainly concentrated in July to August. The rivers in the area include the Yellow River, Nandasha River and Qingshui Gully, among which the Yellow River enters from Pingyin County in the southwest and flows in a northeast direction through the entire area, being the most significant river in the Changxiao hydrogeological unit. The terrain exhibits higher elevations in the southeast and lower topography in the northwest. The geomorphic types are mainly denudation-dissolution low-mid mountain hills and denudation-accumulation intermountain plains. Cultivated land and vegetation coverage are extensively distributed. Cultivated land is mainly concentrated in the relatively flat areas on both sides of the Yellow River, while vegetation is mainly concentrated in the higher terrain in the southeast, and construction land and bare land are dispersed throughout the region.

**Fig 1:**
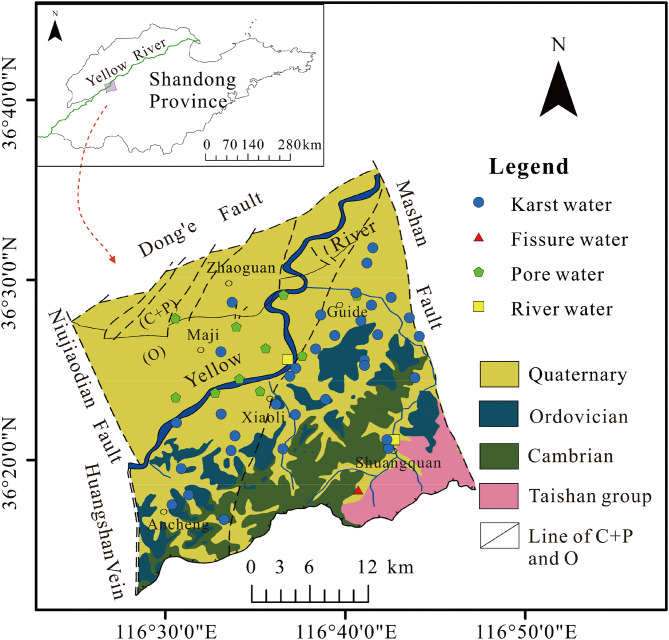
Geological structure of the study area [30] and sampling point distribution map. (Map data comes from Natural Earth. http://www.naturalearthdata.com/).

The Changxiao hydrogeological unit is a relatively independent unit, with its eastern boundary by the Mashan Fault, its southern boundary by the surface watershed, its western boundary by the Huangshan Vein and Niujiaodian Fault, and its northern boundary by the Dong’e Fault (Fig 1). Based on the groundwater storage media, the area primarily develops pore water in unconsolidated rocks, fissure-karst water in carbonate rocks and fissure water in magmatic and metamorphic rocks. The pore water in unconsolidated rocks is mainly distributed on both sides of Nandasha River. In wet season, it is recharged by rainwater and flood seepage of Nandasha River, as well as upward leakage from underlying karst aquifers. Along the Yellow River zone, it is recharged by lateral seepage from the Yellow River. The main discharge modes include runoff discharge, decentralized agricultural extraction and evaporation discharge. Fissure-karst water in carbonate rocks is the main type of groundwater in Changxiao hydrogeological unit, serving as the primary target layer for centralized water supply. Recharge occurs through atmospheric precipitation infiltration, lateral runoff, river lateral infiltration, percolation from overlying porous aquifers, and agricultural irrigation return flow. Discharge is primarily driven by anthropogenic extraction, including decentralized pumping for irrigation and concentrated exploitation at water supply wellfields. Fissure water in magmatic and metamorphic rocks exists in surface weathering fissures, and its water abundance is poor as a whole. It is recharged by atmospheric precipitation, and its discharge is often in the form of descending springs.

### 2.2 Sample collection and analysis

The water sample collection was conducted during the dry season and was completed in June 2022. As shown in Fig 1, a total of 46 samples were collected, including 10 pore water samples, 33 karst water samples, 2 river water samples, and 1 fissure water sample. Groundwater samples were taken from motor wells, with pore water holes drilled at depths of 15.2–63.5 m, karst water holes drilled at depths of 60–563.1 m, and fissure water holes drilled at depths of 97–236 m. Before sampling, sample bottles were cleaned with distilled water. During sampling, each bottle was rinsed three times with extracted well water. The water was pumped for 5–10 minutes before collection. At the same time, some parameters were quickly tested on site to ensure sample integrity and representativeness. After recording the sampling location and time characteristics, the samples were sent to the laboratory of Shandong Provincial Geo-mineral Engineering Exploration Institute for testing. K^+^, Na^+^, Ca^2+^ and Mg^2+^ were determined by inductively coupled plasma atomic emission spectrometer (ICP-OES, OPTIMA 7000DV), while Cl^-^, SO_4_^2-^ and NO_3_^-^ were determined by CIC-D120 ion chromatograph, and HCO_3_^-^ and TDS were determined by EDTA titration. To verify the accuracy of the test results, all hydrochemical test results were calculated using the ion balance error (E). The ion balance error (E) of all samples was within the acceptable range of ± 10% [31,32], meeting the analysis requirements. The field investigation and sample collection conducted in this study did not involve any protected areas, endangered species, or areas subject to special legal restrictions. All sampling points are located on public land or open areas, and the research activities fully comply with local environmental protection regulations. Therefore, no additional permits or approvals are required for the field investigation and sample collection in this study.

### 2.3 Nitrate pollution index (NPI)

Nitrate pollution index was used to measure the pollution degree of nitrate in groundwater. Nitrate was the most common pollutant artificially introduced into groundwater. According to the “Standard for groundwater quality” of China (GB/T 14848−2024), centralized domestic water can be used when the concentration of nitrate (calculated as nitrogen) in groundwater does not exceed 20 mg/L. When it exceeds 20 mg/L, the pollution and health risks caused by nitrate should be considered. The NPI calculation formula can be expressed as [11]:


$$\begin{array}{c} NPI=\frac{Cs-HAF}{HAF} \end{array}$$ (1)

Where *Cs* is the concentration of NO_3_^-^-N; *HAF* is the background concentration of nitrate in groundwater, which is considered an acceptable value for humans. Here, the Class III water standard limit (20 mg/L) in the “Standard for groundwater quality” of China (GB/T 14848−2024) is taken.

NPI was an important indicator to describe the degree of nitrate pollution in groundwater. Generally, the larger the NPI, the more serious the nitrate pollution. According to the NPI value, as shown in Table 1, groundwater was divided into five categories [11,33–35].

**Table 1:** Classification of nitrate pollution index.

Value of NPI	Pollution degree
< 0	Clean
0-1	Light
1-2	Moderate
2-3	Significant
> 3	Very significant

### 2.4 Multivariate statistical analysis

#### 2.4.1 Data processing.

11 hydrochemical indicators including pH, TH, TDS, Ca^2+^, Mg^2+^, K^+^, Na^+^, Cl^-^, HCO_3_^-^, SO_4_^2-^ and NO_3_^-^-N were selected for multivariate statistical analysis. When the detection value of chemical ions was lower than the detection limit (DL), DL/2 was used to replace the value [36]. Each parameter should be evaluated for its normality (i.e., Gaussian distribution) to determine suitability for multivariate statistical analysis. In this study, Johnson transformation was applied to each non-normally distributed parameter, selecting the best fit with a p value of 0.1 to transform the data into normal distributions [17]. To ensure equal weights and eliminate the influence of dimensionality, the Z-score was calculated for standardization [37].

$$\begin{array}{c} Z_{i}=\frac{X_{i}-\overset{―}{X}}{\sigma } \end{array}$$ (2)

Where *Z*_*i*_ is the standard score of sample i; *X*_*i*_ is the value of sample i; *X* is the average value and σ is the standard deviation.

#### 2.4.2 Hierarchical cluster analysis.

Hierarchical cluster analysis was used to analyze multidimensional hydrochemical data sets. This method classifies water samples into distinct groups based on variable similarities, while also enabling the grouping of chemical components according to their sources [38,39]. In this study, hierarchical cluster analysis was used to classify the samples based on their hydrochemical characteristics, elucidating the distinct hydrogeochemical features and potential interrelationships among different water groups. Ward’s linkage method and square Euclidean distance were used as similarity measures [38], and the classification results were displayed in the form of a tree. Simultaneously, one-way analysis of variance (ANOVA) test was used to evaluate the clustering effect. ANOVA can be used to compare the means of three or more groups of data to determine if there is a significant difference. The F value reflects the ratio of inter group variation to intra group variation, and the larger the value, the more likely the inter group difference is to be significant. When the significance level P is less than 0.05, it is considered that there are at least two groups with significantly different means. Afterwards, multiple comparisons can be made pairwise to further clarify which two categories have significant differences.

#### 2.4.3 Factor analysis.

Factor analysis was a dimensionality reduction method, which used a few factors to describe the potential relationship between many variables. In this study, factor analysis was used to identify the potential factors affecting the hydrogeochemical characteristics of groundwater. The maximum likelihood method was selected as the factor selection method, and the maximum variance method was the rotation method [17]. Kaiser-Meyer-Olkin (KMO) > 0.6 indicated that the dataset was considered suitable for factor analysis, and the factors were extracted based on the eigenvalue > 1 [21]. The importance of variables to factors were determined based on factor loading magnitudes, while factor scores were retained to quantify the influence of factors on water samples. Factor analysis combined with hierarchical cluster analysis was used to analyze the factors affecting groundwater hydrogeochemistry from the perspectives of variables and cases, and to clarify the influence degree and characteristics of nitrate.

### 2.5 Probabilistic health risk assessment

#### 2.5.1 Human health risk assessment.

Human health risk assessment can effectively assess the relationship between harmful substances in the water environment and human health. This paper adopted the health risk method provided by the USEPA to evaluate the health risk posed by nitrate in groundwater to different age groups [40]. The assessment model included four parts: risk identification, dose effect analysis, exposure assessment and risk characterization [7,41,42]. This study focuses on the potential harm of groundwater nitrate to human health. Given the significant differences in sensitivity to pollutants among populations with different physiological characteristics and behavioral patterns, the study divided the target population into two age groups: children and adults. Focusing on the two main exposure pathways of drinking ingestion and skin contact, the study delved into the risk level of nitrate on human health [7]. Health risk assessment can be expressed as [43,44]:


$$\begin{array}{c} {CDI}_{oral}=\frac{C\times IR\times {EF}_{oral}\times ED}{BW\times AT} \end{array}$$ (3)

$$\begin{array}{c} {CDI}_{dermal}=\frac{C\times ET\times K_{p}\times SA\times {EF}_{dermal}\times ED\times CF}{BW\times AT} \end{array}$$ (4)

$$\begin{array}{c} {HQ}_{oral}=\frac{{CDI}_{oral}}{{RfD}_{oral}} \end{array}$$ (5)

$$\begin{array}{c} {HQ}_{dermal}=\frac{{CDI}_{dermal}}{{RfD}_{dermal}} \end{array}$$ (6)

$$\begin{array}{c} HQ={HQ}_{oral}+{HQ}_{dermal} \end{array}$$ (7)

Nitrate is a non-carcinogenic harmful substance. Therefore, the hazard quotient (*HQ*) was employed to assess its potential non-carcinogenic risks to human health [45,46]. USEPA gives a *HQ* threshold of 1, *HQ* < 1 indicates the risk of non-carcinogenic effects on health caused by nitrate pollution in groundwater within the acceptable range, and *HQ* > 1 indicates the risk of non-carcinogenic effects on health beyond the acceptable range [21,40]. *CDI*_*oral*_ and *CDI*_*dermal*_ are the average daily intake (mg/(kg·d)) calculated by oral and skin exposure routes respectively; *C* is the content of nitrate in groundwater (mg/L); *RfD* is the reference dose (mg/kg/d); *IR* is the intake rate, measured in L/d; *EF* is the exposure frequency (d/y); *ED* is the exposure period (y); *BW* is body weight (kg); *AT* is the average lifespan (d); *ET* is the exposure time (h/d)；*SA* is the skin surface area (cm^2^), *Kp* is the skin penetration coefficient (cm/h).

#### 2.5.2 Monte Carlo simulation.

In human health risk assessment, parameters were usually assigned specific values [46,47], which brought great uncertainty to the assessment results. To solve this problem, many scholars used Monte Carlo simulation to conduct probabilistic risk assessment to reduce the uncertainty of risk [48]. Monte Carlo simulation was a powerful probabilistic risk analysis method, which used random number generation and iterative operation to simulate the occurrence of risk events, and expressed the results as probability distribution [49]. In specific problems, hundreds and thousands of simulations were usually used to obtain the probability of each event or the arithmetic mean of the observed values of each event, so as to understand the solution of the problem [50]. In this paper, Crystal Ball software was used for Monte Carlo simulation to obtain the distribution characteristics of HQ in children and adults, so as to more accurately reflect the potential risk of groundwater to human health. When the number of Monte Carlo simulation increased to 10000, it ensured that the risk in the process of random generation was consistent at 95% [51].

Therefore, the values and distribution characteristics of various exposure parameters of the probabilistic health risk assessment are shown in Table 2.

**Table 2:** Parameter values and distribution characteristics for probabilistic health risk assessment [52–54].

Parameter	Unit	Pathway	Value		Distribution
			Children	Adult	
*C*	mg/L	Oral/dermal	Measured	Measured	Gamma
*IR* (ingestion rate)	L/d	Oral	(0.85,0.09)	(1.5,0.15)	Normal
*EF* (exposure frequency)	d/y	Oral	365	365	
		Dermal	(180,345,365)	(180,345,365)	Triangular
*ED* (exposure duration)	y	Oral/dermal	(0,6)	(18,50)	Uniform
*BW* (body weight)	kg	Oral/dermal	(15,1.5)	(61.75,6.18)	Log-normal
*AT* (average lifespan)	d		ED*365		
*ET* (exposure time)	h/d	Dermal	0.33	0.25	
*SA* (skin surface area)	cm^2^	Dermal	(5838,920)	(19771,3373)	Log-normal
*Kp* (skin penetration coefficient)	cm/h		0.001	0.001	
*CF*	L/cm^3^		0.001	0.001	
*RfD*	mg/kg/d		1.6	1.6	

## 3 Results

### 3.1 General hydrochemical characteristics of groundwater

**Fig 2:**
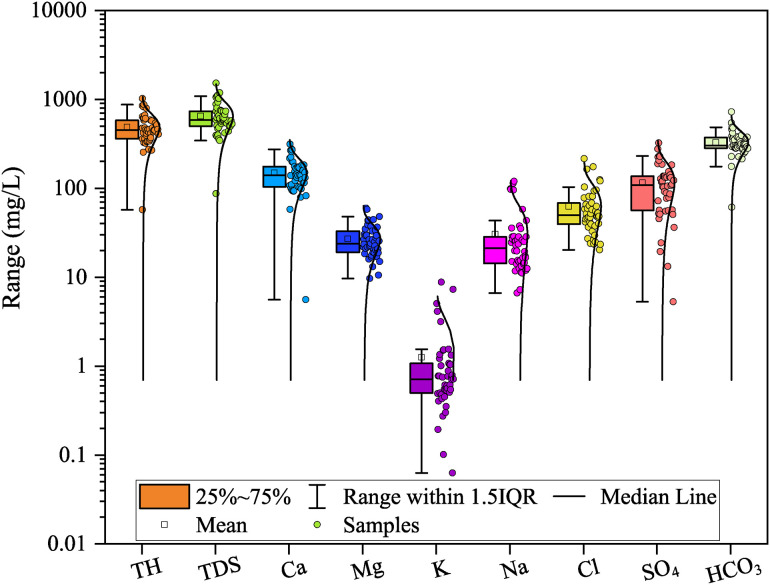
The value distribution of all hydrochemical parameters.

The statistical data of hydrochemical parameters of groundwater and river water samples in the study area are shown in Fig 2 and Table 3. Both groundwater and river water are weakly alkaline, with pH values ranging from 7.22 to 8.18. On average, the alkalinity of fissure water is the strongest, followed by river water, while pore water and karst water display similar pH levels. In groundwater, Ca^2+^ is the dominant cation, among which pore water samples exhibit the highest Ca^2+^ content, followed by karst water. According to average mass concentrations, the order of cations in pore water is Ca^2+^ > Na^+^ > Mg^2+^ > K^+^, while the order in fissure water and karst water is Ca^2+^ > Mg^2+^ > Na^+^ > K^+^. HCO_3_^-^ is the dominant anion in groundwater, with the highest contents found in pore water, then karst water. According to average mass concentrations, the order of anions in groundwater is HCO_3_^-^> SO_4_^2-^ > Cl^-^. In river water, Na^+^ content is higher than other cations, which is the dominant cation, followed by Ca^2+^. The content of SO_4_^2-^ is higher than other anions, which is the dominant anion, followed by HCO_3_^-^.

**Table 3:** Statistics of groundwater and river water chemical index values.

Parameters	Pore water			Karst water			River water			Fissure water
	Min	Max	Mean	Min	Max	Mean	Min	Max	Mean	
pH	7.24	8.12	7.59	7.22	8.18	7.55	7.71	7.95	7.83	7.99
TH	320.11	1017.59	522.44	57.22	871.51	485.17	254.04	356.98	305.51	392.19
TDS	384.31	1514.87	716.19	86.99	1179.36	631.77	586.01	735.74	660.88	482.58
Ca^2+^	92.4	312	155.43	5.6	275	153.11	57.7	107	82.35	92.9
Mg^2+^	18.3	59.6	32.61	9.64	47.9	24.97	21.8	26.7	24.3	38.9
Na^+^	14.9	120	50.65	6.62	57.7	20.29	96.9	98.7	97.8	25.2
K^+^	3.15	0.19	0.99	0.063	7.26	0.90	4.13	5.03	4.58	8.76
Cl^-^	27	215	77.28	20.3	174	57.03	77.6	103	90.3	36.4
SO_4_^2-^	19.3	323	99.46	5.29	231	113.18	188	276	232	118
HCO_3_^-^	295	721	414.9	60.9	487	311.44	176	228	202	286

### 3.2 Nitrate pollution characteristics

**Fig 3:**
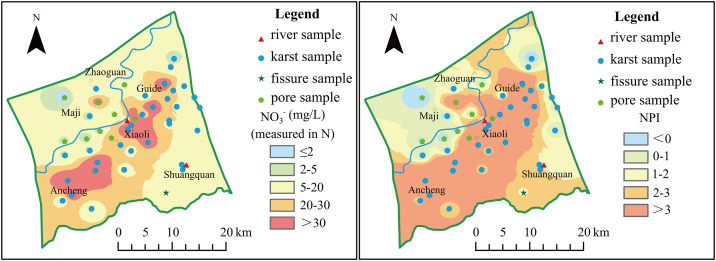
Distribution of NO_3_^-^-N concentrations and NPI values. (Map data comes from Natural Earth. http://www.naturalearthdata.com/).

Table 4 presents the statistical characteristics of NO_3_^-^-N in different water samples. The average concentration of NO_3_^-^-N is 19.48 mg/L, and the maximum concentration is 68.42 mg/L. Among them, 34.78% of water samples had NO_3_^-^-N content exceeding the Class III groundwater quality standard limit (20 mg/L as N) in “Standard for groundwater quality” of China (GB/T 14848−2024). Among the groundwater samples, the pore water exhibits the highest average nitrate concentration, followed by karst water, while fissure water is the lowest levels. Compared to groundwater, the NO_3_^-^-N content in river water is significantly lower. The standard deviation (SD) and coefficient of variation (CV) reflect the degree of dispersion and variability of data. The pore water and karst water show higher SD and CV values, indicating a wide variation range in NO_3_^-^ concentrations and significant spatial distribution differences. Fig 3 shows the distribution of NO_3_^-^-N content in the study area. It can be seen that the nitrate content of stations near Xiaoli Town, Guide Town and Ancheng Town is far higher than that of other places. In contract, regions along and north of the Yellow River exhibit lower nitrate levels, attributable to lateral runoff recharge [27] and riverbank infiltration from the Yellow River diluting nitrate concentrations. Nitrate accumulation is primarily concentrated in areas with intensive human activities, where elevated nitrate levels correlate strongly with high population density.

**Table 4:** Statistical characteristics of NO_3_^-^-N in water samples of the study area.

Type	Max (mg/L)	Min (mg/L)	Mean (mg/L)	SD	CV (%)	Over-standard rate (GB/T14848-2024) (%)
Permissible limit						20 mg/L
Karst water	57.81	ND	20.11	16.50	82.05	36.4
Pore water	68.42	ND	21.06	26.61	126.35	40
Fissure water	–	–	13.07	–	–	0
River water	5.83	3.07	4.45	1.95	43.82	0
Total	68.42	ND	19.48	18.63	95.64	34.78

ND means not detected, and the value is lower than the detection limit.

**Fig 4:**
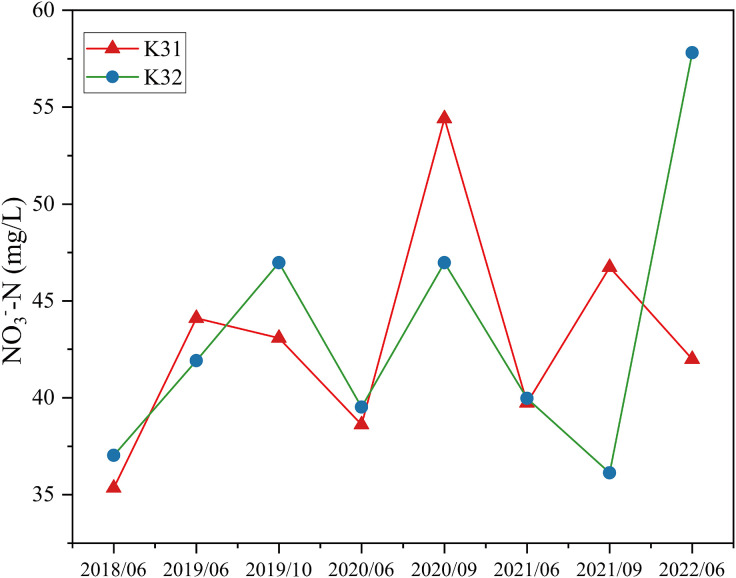
Multi-year variation characteristics of K31 and K32 NO_3_^-^-N.

Meanwhile, the study selected NO_3_^-^-N concentration data from multiple years at two points, K31 (Xixin Village, Xiaoli Town) and K32 (Guangli Village, Xiaoli Town), to analyze the multi-year variation characteristics of nitrate (Fig 4), which were located in the groundwater discharge area. The aquifers are dolomite and limestone of the Cambrian-Ordovician Sanshanzi Formation-Majiagou Group. The data were sourced from June 2018–2022, October 2019, and September 2020 and 2021, respectively. June is the dry season in the study area, while September and October are the wet season.

As shown in Fig 4, the nitrate content at point K31 and point K32 has fluctuated, but the concentrations have remained above 20 mg/L for many years, indicating a serious pollution situation. The two points exhibit similar trends in nitrate concentration variation. From 2018 to 2020, nitrate concentration first increased and then decreased, reaching a minimum in June 2020, after which increased rapidly, especially at K31 where the NO_3_^-^-N reached the maximum in September 2020. After 2021, the NO_3_^-^-N concentrations at point K31 and point K32 showed opposite changes. At point K32, the nitrate concentration continued to decline in 2021, but increased sharply in 2022. At point K31, the concentration still fluctuated.

At the same time, it can be seen that compared with the changes in NO_3_^-^-N concentration during the wet season and dry season, the content of NO_3_^-^-N during the dry season is relatively low. This may be because there is less precipitation during the dry season, resulting in less nitrate from human activities infiltrating into groundwater. Meanwhile, groundwater flow rates are lower in the dry season, and since the samples are located in the discharge area, pollution transported from upstream regions is also less.

### 3.3 Nitrate pollution index

NPI is used to measure the pollution degree of nitrate in groundwater, and it is also one of the indicators to evaluate groundwater quality. In the study area, the NPI values ranged from −0.992 to 14.15, with an average of 3.31. According to the NPI classification of water samples in Table 5, about 28.26% of the samples are clean, 10.87% of the water samples are slightly polluted, 10.87% of the water samples are moderately polluted, and the remaining 50% of the water samples are seriously polluted by nitrate.

**Table 5:** NPI classification of water samples in the study area.

Value of NPI	Number of pore samples	Number of karst samples	Number of fissure samples	Number of river samples	All number	%
< 0	5	7		1	13	28.26
0-1	1	3		1	5	10.87
1-2		4	1		5	10.87
2-3		4			4	8.70
> 3	4	15			19	41.30

The distribution of NPI and NO_3_^-^-N content is consistent (Fig 3). The areas with serious groundwater pollution are mainly located near Guide Town, Xiaoli Town and Ancheng Town. The pollution is distributed in the northeast-southwest direction, which is more serious in the southern part of the Yellow River.

### 3.4 Results of hierarchical cluster analysis

**Fig 5:**
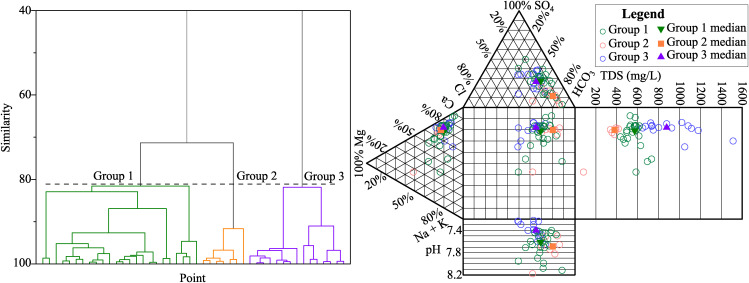
Tree diagram of hierarchical cluster analysis and Durov diagram of different groups of water chemistry.

Hierarchical cluster analysis was applied to the study of groundwater chemistry characteristics. The water samples were divided into different groups to analyze the similarities and differences and show the hydrochemical characteristics in different groups (Fig 5). In this study, all water samples were divided into three groups, and the similarity of each point between groups was more than 80%. The ANOVA test was conducted on the clustering results, and the results showed (Table 6) that except for K^+^, there were significant differences (P < 0.05) in all other ions among the three types of water samples. Among them, the inter group differences of TH, TDS, Ca^2+^ and NO_3_^-^-N were the most significant, indicating that cluster analysis effectively divided water samples with different hydrochemical characteristics and pollution levels. And LSD multiple comparisons were conducted for each clustering group, and the comparison results are also shown in Table 6. Compared with cluster 2 and cluster 1, it shows significant differences in all ions except for pH, K^+^ and HCO_3_^-^. Compared with cluster 3, there are significant differences between ions in cluster 2 (except for K^+^). There is no significant difference in K^+^ and Na^+^ between cluster 1 and cluster 3, while there are significant differences in other ions. There are significant differences among the groups in terms of mineral dissolution and human activities.

**Table 6:** ANOVA results for ions and LSD multiple comparison results.

		pH	TH	TDS	Ca^2+^	Mg^2+^	Na^+^	K^+^	Cl^-^	SO_4_^2-^	HCO_3_^-^	NO_3_^-^-N
F		16.04	76.63	89.12	48.97	10.90	3.75	0.98	21.58	17.31	4.77	42.94
P	P	0.000	0.000	0.000	0.000	0.000	0.032	0.383	0.000	0.000	0.013	0.000
	1, 2	0.403	0.000	0.000	0.000	0.036	0.037	0.737	0.005	0.000	0.440	0.004
	2, 3	0.000	0.000	0.000	0.000	0.000	0.009	0.495	0.000	0.000	0.012	0.000
	1, 3	0.000	0.000	0.000	0.000	0.002	0.334	0.169	0.000	0.000	0.012	0.000

Table 7 shows the average values of chemical parameters for clustered water samples. Group 1 (G1) contains 24 samples, including river water samples, pore water samples, karst water samples and fissure water samples. The hydrochemical type of G1 is mainly HCO_3_-Ca type, partially affected by SO_4_^2-^. The parameter values of G1 are all at moderate levels among three sets of water samples, fully displaying the geological and hydrogeological characteristics. Dolomite and limestone are the main aquifers for groundwater storage in the study area, mainly composed of dolomite and calcite, accompanied by gypsum, salt rock and other minerals [28]. During groundwater migration, water-rock interactions lead to the leaching and dissolution of minerals within the aquifer, forming a hydrochemical field with lithological characteristics. The differential dissolution of calcite and dolomite dominates the distribution of Ca^2+^, Mg^2+^ and HCO_3_^-^, while the presence of gypsum and salt rock further changes the groundwater dissolution balance. At the same time, the average value of NO_3_^-^-N content in this group is in a relatively safe range, but still some samples where NO_3_^-^-N content exceeds the standard, indicating that human activities have an impact on G1. The dominant processes controlling the hydrogeochemical characteristics of G1 are carbonate rock weathering and trace anthropogenic inputs. River water samples, fissure water samples and some pore water samples are grouped with karst water samples, indirectly indicating the hydraulic connections between them. However, compared with other water samples, river water and pore water have relatively high Na^+^ and Cl^-^ contents, indicating more pronounced effects of evaporation and concentration.

**Table 7:** The mean values of physicochemical parameters in the hierarchical cluster analysis groups of the study area (unit: mg/L, except pH).

Group	n	pH	TH	TDS	Ca^2+^	Mg^2+^	K^+^	Na^+^	Cl^-^	SO_4_^2-^	HCO_3_^-^	NO_3_^-^-N
1	24	7.66	418.35	565.41	126.55	24.85	1.48	31.23	50.33	110.61	321.65	10.40
2	7	7.72	273.53	339.88	80.74	17.46	0.74	14.1	30.73	33.66	269.7	2.73
3	15	7.39	685.54	924.39	217.47	34.59	1.13	36.58	96.59	161.41	367.28	41.83

Group 2 (G2) contains 7 water samples. The main anion is HCO_3_^-^, while the main cation is Ca^2+^, with the hydrochemical type of HCO_3_-Ca type, which is typical karst groundwater in carbonate rocks and is affected by water-rock interaction. Compared with the other two groups, the values of hydrochemical parameters are at a low level. Among them, TH and TDS are one of the indicators for judging the quality of water. The lower TH and TDS values of G2 indicate that its water quality is good and least affected by human activities. Group 3 (G3) contains 15 water samples, with the predominant hydrochemical type remaining as HCO_3_-Ca type, while some samples show influences from SO_4_^2-^ and Cl^-^. The content of TH and TDS in G3 is the most, indicating poor water quality with high levels of chemical ions. Among them, the content of NO_3_^-^-N is much higher than the other two groups, and far exceeds the Class III water standard limit (20 mg/L). Human activities are one of the reasons for nitrate exceeding the standard, seriously altering the original hydrochemical field. G3 represents anthropogenically impacted groundwater exhibiting contamination characteristics.

### 3.5 Results of factor analysis

**Fig 6:**
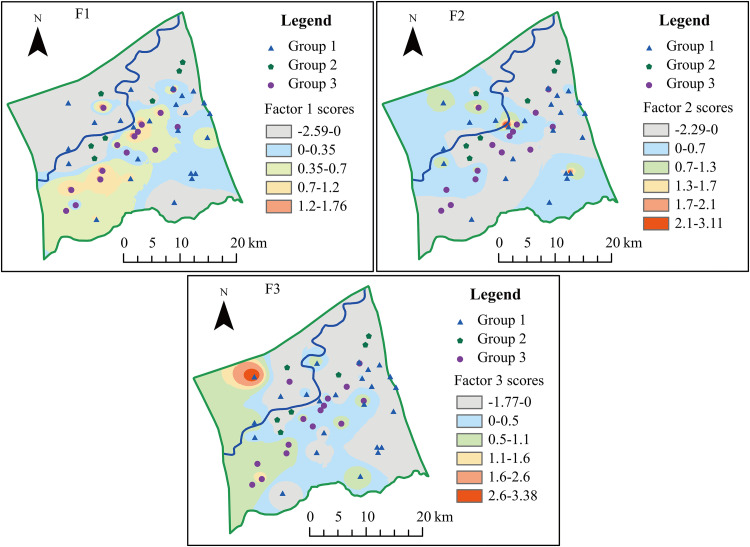
Distribution of factors influence and cluster groups. **(a)** Facotr 1; **(b)** Factor 2; **(c)** Factor 3 (Map data comes from Natural Earth. http://www.naturalearthdata.com/).

The results of factor analysis are shown in Table 8. The size of factor loadings reflects the influence of variables on factors. For the convenience of analysis, only factor loadings > 0.5 are presented in the table. The factor score of each sample is related to the process or source described by each factor. When the factor score is negative, it indicates that the performance of the water sample in the hydrochemical characteristics represented by the corresponding factor is lower than the average level; conversely, when the factor score is positive, it represents that the performance in that factor is above the average level. Fig 6 shows the interpolation diagram of factor scores of each sample to show the spatial influence range of each factor and the distribution of cluster groups.

**Table 8:** Factor loadings of groundwater chemical parameters (factor loadings below 0.5 are not provided for simplicity of analysis).

Variable	F1	F2	F3
pH	−0.765		
TH	0.806		0.587
TDS	0.770		
Ca^2+^	0.923		
Mg^2+^			0.779
K^+^		0.559	
Na^+^		0.779	
Cl^-^	0.541	0.646	
SO_4_^2-^	0.655		
HCO_3_^-^			0.703
NO_3_^-^-N	0.858		
Eigenvalue	5.910	2.046	1.215
% of Variance	53.731	18.604	11.045
Cumulative %	53.731	72.335	83.380

**Fig 7:**
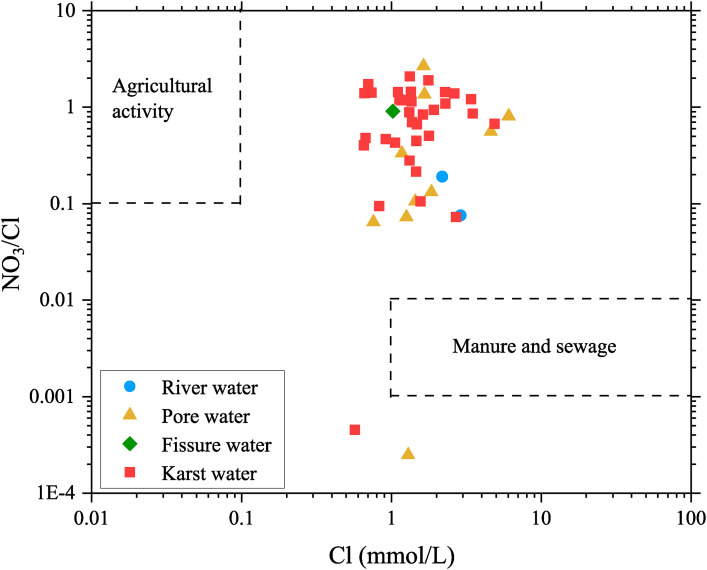
Scatter diagram of NO_3_/Cl vs Cl.

According to Table 8, three main factors with eigenvalues > 1 are extracted, and the cumulative variance interpretation rate reaches 83.38%, which can effectively explain the main factors affecting groundwater chemistry characteristic in the study area and analyze the main reasons for NO_3_^-^-N exceeding the standard. Factor 1 (F1), the most influential factor, contributes 53.731% to the hydrogeochemical characteristics of the study area. TH, TDS, Ca^2+^ and NO_3_^-^-N exhibit significantly high positive loadings on F1, while Cl^-^ and SO_4_^2-^ have moderate positive loadings. Ca^2+^ is the most important cation in groundwater, which mainly comes from the dissolution of rocks and minerals. Combined with the geological background, high positive loadings of TH, TDS and Ca^2+^ reflect the weathering and dissolution of limestone and gypsum. Meanwhile, based on the correlation matrix (Table 9), Ca^2+^ and NO_3_^-^ have a strong positive correlation, while Cl^-^ and SO_4_^2-^ also exhibit a certain degree of positive correlation with NO_3_^-^-N. In addition to the dissolution of Ca^2+^, Cl^-^ and SO_4_^2-^ through water-rock interaction, there are other sources contributing to their presence. Nitrate reflects the impact of human activities to a certain extent [55]. The study area contains extensive cultivated lands, and the existence of agricultural activities increases the content of nitrate in groundwater. Overapplication of nitrogen fertilizers exceeding the capacity of plant roots leads to surplus soil nitrogen, which is subsequently transported to groundwater via infiltration under precipitation and irrigation. The use of common fertilizers such as (NH_4_)_2_SO_4_ and CaCO_3_ not only increases nitrate concentrations, but also increases the concentration of corresponding ions, thereby altering the original hydrochemical field. Cl^-^ is stable and an ideal tracer for water pollution. At the same time, different NO_3_^-^ sources exhibit different NO_3_^-^/Cl^-^ levels, so that the molar ratio range of NO_3_^-^/Cl^-^ can help identify the source of nitrate pollution [56]. Fig 7 shows the distribution characteristics of samples based on NO_3_/Cl vs Cl plot. Most samples fall within the range of Cl^-^ > 1 mmol/L and 0.1 < NO_3_^-^/Cl^-^ < 10, indicating that sewage, manure and agricultural activities collectively contribute to elevated nitrate concentrations in groundwater. In conclusion, F1 can be interpreted as the dissolution of minerals and the impact of human activities, with the influence of human activities being relatively significant.

**Table 9:** Correlation matrix in factor analysis.

parameter	pH	TH	TDS	Ca	Mg	K	Na	Cl	SO_4_	HCO_3_	NO_3_
pH	1	−0.666	−0.594	−0.754	−0.151	0.303	−0.082	−0.452	−0.409	−0.385	−0.587
TH		1	0.920	0.959	0.605	−0.276	0.255	0.595	0.589	0.644	0.821
TDS			1	0.862	0.618	−0.102	0.473	0.754	0.723	0.470	0.792
Ca				1	0.399	−0.324	0.151	0.545	0.600	0.564	0.867
Mg					1	0.107	0.506	0.539	0.400	0.537	0.369
K						1	0.418	0.098	0.256	−0.357	−0.180
Na							1	0.741	0.370	0.172	0.158
Cl								1	0.585	0.218	0.551
SO_4_									1	0.007	0.631
HCO_3_										1	0.264
NO_3_											1

The contribution rate of factor 2 (F2) to hydrogeochemistry in the study area is 18.604%. Na^+^ has a high positive loading, while K^+^ and Cl^-^ exhibit moderate positive loadings. Na^+^ and K^+^ mainly come from the dissolution of silicate minerals and evaporite minerals, while evaporative concentration further enhances the enrichment of Na^+^ and Cl^-^. In F2, Na^+^ and Cl^-^ account for the main loading, indicating that in areas with shallow groundwater tables or direct exposure of limestone, hydrochemistry is strongly controlled by evaporative concentration, thus showing the characteristics that Na^+^ and Cl^-^ mainly control hydrochemistry. The contribution rate of factor 3 (F3) to hydrogeochemical characteristics is 11.045%. Mg^2+^ and HCO_3_^-^ exhibit high positive loadings. The groundwater in this area is mainly karst water, with the karst aquifer system primarily composed of Cambrian and Ordovician carbonate formations, where the lithology consists mainly of limestone and dolomite. F3 mainly reflects the dissolution of dolomite.

Fig 6 shows the distribution of factor scores for each sample, with comparative analysis based on hierarchical cluster grouping. The regions with high F1 scores are mainly distributed in the south bank of the Yellow River, and coincide with all samples of G3 and some samples of G1. The areas with severe nitrate pollution have higher F1 scores, indicating the main influence of human activities. The high F2 score areas are mainly distributed in the northwest and southeast of the study area, with the largest impact at two river water samples, indicating that the river is more obviously affected by evaporative concentration. Meanwhile, in the alluvial plains on both sides of the Yellow River, the shallow groundwater depth makes the hydrochemical characteristics influenced by evaporative concentration. High F3 scores are mainly distributed in the west of the study area, covering some samples of G1 and G3. Thus, G1 and G3 coincide with the high score areas of F1, F2 and F3. The most samples have created a unique hydrochemical field under the joint influence of a variety of factors. Especially, G3 represents samples primarily influenced by anthropogenic activities, which is still affected by natural factors. Although G2 has typical hydrochemical characteristics of karst water, due to the presence of human activities and evaporation and concentration, which alter the natural chemical characteristics of the water, it shows negative scores in all three factors, and its hydrochemical index is lower than the overall average level of the study area.

### 3.6 Results of probabilistic health risk assessment

From the perspective of human health, drinking groundwater containing high nitrate can have adverse effects on human health. Children, especially infants, are more likely to suffer from methemoglobinemia due to enzyme activity levels not reaching normal levels, which can also lead to imbalances in blood sugar and thyroid hormones in the body [9,57,58]. In this study, Monte Carlo simulation was used to assess health risks of nitrate in different intake routes. The traditional deterministic risk assessment brought great uncertainty to health risk. Monte Carlo simulation used the probability distribution of exposure parameters to reduce the occurrence of uncertainty. Karst water serves as the primary aquifer for water supply, with pore water infiltration acting as one of its recharge sources. Given the hydraulic connection between two systems, this study evaluated the potential risks of nitrate contamination in both karst and pore water to assess human health risks posed by groundwater. The fitting distribution in Crystal Ball software was used to judge that the nitrate data in karst water conformed to gamma distribution, and the nitrate data in pore water was verified that Gamma distribution could not be rejected. Therefore, Gamma distribution was adopted for the nitrate concentration data in the Monte Carlo simulation. And increasing the number of simulations to 10000 to ensure that the risk is consistent with a 95% probability during the random generation process.

**Fig 8:**
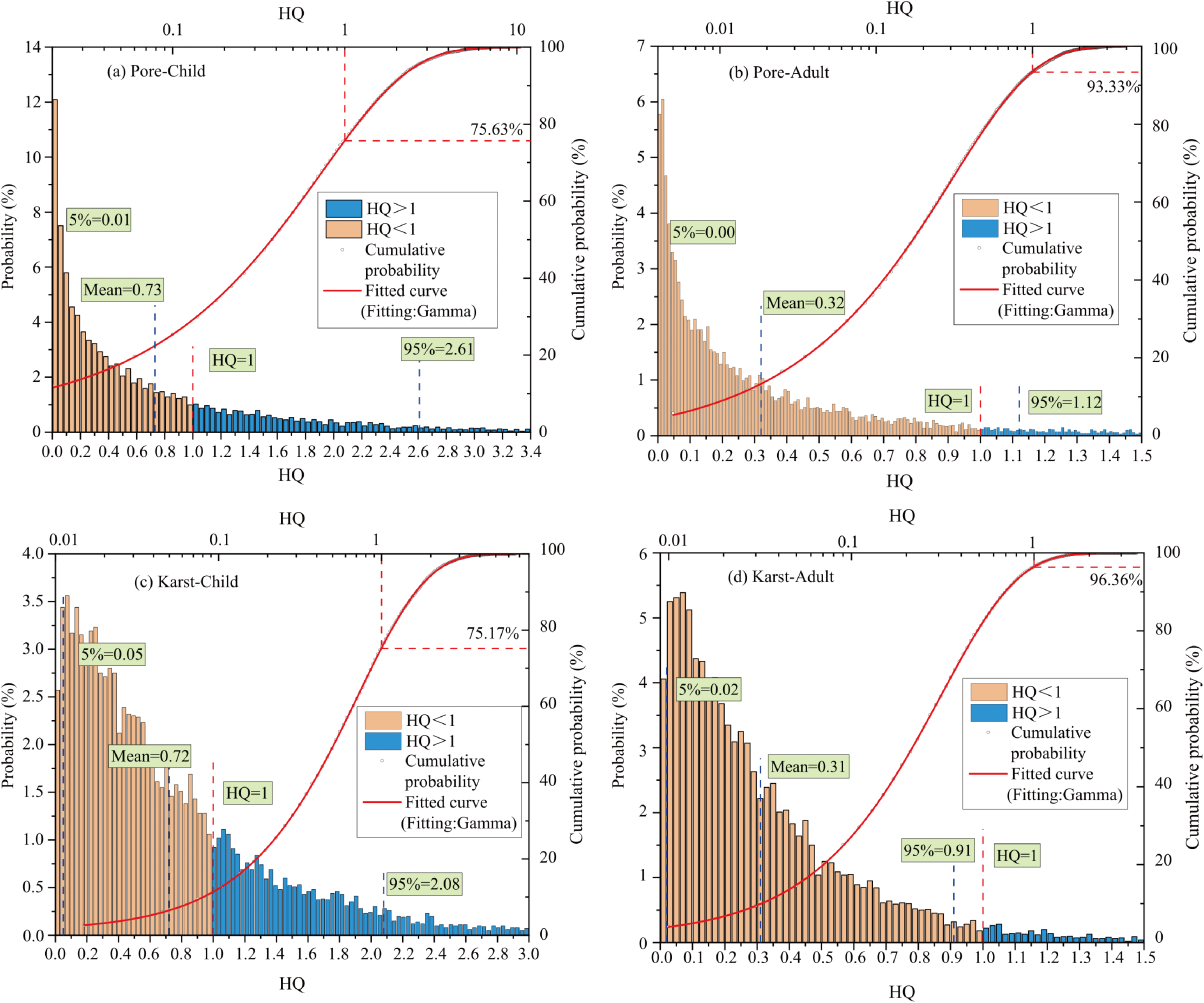
Monte Carlo simulation results for different age groups. **(a)** Pore-Child; **(b)** Pore-Adult; **(c)** Karst-Child; **(d)** Karst-Adult.

Fig 8 presents risk histograms and cumulative probability distribution curves of pore water and karst water for children and adults respectively. USEPA sets the threshold to 1 and states that when the HQ value is below 1, even sensitive individuals are unlikely to suffer from the adverse health effects of this chemical substance [59]. As shown in Fig 8a, 8b, the average HQ values of children and adults in pore water are 0.73 and 0.32, respectively, which are lower than the threshold value 1. However, the HQ values at the 95^th^ percentile are 2.61 and 1.12, respectively, indicating that nitrate in pore water has an unacceptable non-carcinogenic risk for children and adults. Meanwhile, the probability distribution shows that the probability of exceeding the threshold 1 was 24.37% and 6.67% for children and adults respectively, indicating children face a higher probability of nitrate exposure and greater non-carcinogenic risks compared to adults. Fig 8c and 8d respectively show the potential risk distribution of nitrate in karst water to children and adults. The average HQ values of children and adults are 0.72 and 0.31, respectively, and HQ values at the 95^th^ percentile are 2.08 and 0.91, respectively. The probability of exceeding the threshold 1 was 24.83% and 3.64% for children and adults, respectively. In karst water, the potential risk of nitrate to adults is far less than that to children who should be paid more attention. Compared the non-carcinogenic risk levels of nitrate in karst water and pore water, from the perspective of probability distribution, adults are more likely to be harmed by nitrate in pore water, while children have little difference.

## 4 Discussion

### 4.1 Driving factors of nitrate pollution

The geological background dominates the spatial differentiation and accumulation characteristics of nitrate pollution. Different lithological units have a significant controlling effect on the enrichment and migration of nitrate ions. The concentration of nitrate in loose rock pore water is generally higher than that in karst water and fissure water. Loose rocks are composed of alluvial deposits with high permeability, which facilitate vertical infiltration of atmospheric precipitation and irrigation water, and can carry nitrate produced by surface agricultural activities into groundwater. Loose rocks contain a large amount of clay minerals, and their adsorption capacity for nitrate is limited. Nitrate ions are easily migrated with water flow and are not easily blocked. The spatial distribution of nitrate shows significant heterogeneity. Carbonate rock fissure karst water is the main type of groundwater in the study area. The carbonate rock fissure karst water bearing rock formations are mainly composed of Cambrian-Ordovician limestone and dolomite, with significant differences in water yield and strong heterogeneity and anisotropy. The southeastern mountainous areas are exposed to carbonate rocks, with developed valleys and surface karst, which is highly conducive to the infiltration of surface water and serves as a recharge area for karst water. The carbonate rocks in the Xiaoli-Guide area are hidden beneath the Quaternary system, with well-developed fissure karst and strong water richness. The nitrate migrated upstream and the nitrate input by local people converge here, forming a high pollution area. At the same time, under the influence of water rock interaction, HCO_3_^-^ neutralizes the H^+^ produced by nitrification during the dissolution process of carbonate rocks, inhibits the reduction reaction of NO_3_^-^, and allows it to exist stably in groundwater for a long time, exacerbating pollution accumulation. The fissure water of igneous and metamorphic rocks is mainly buried in the network weathering fissure zone of igneous rocks, with poor permeability and difficulty in infiltrating large amounts of surface pollutants. It is less affected by external human input, and the fissure water in the study area is distributed in the southeast recharge area, with low nitrate concentration in groundwater.

**Fig 9:**
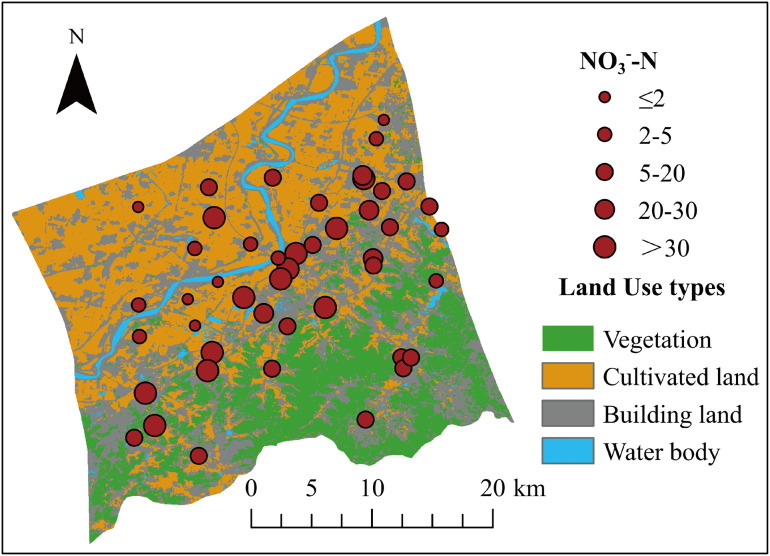
Distribution of nitrate contamination in relation to land use types. (The boundary data comes from Natural Earth. http://www.naturalearthdata.com/; Land use data comes from USGS EROS (Earth Resources Observatory and Science (EROS) Center) (public domain). http://eros.usgs.gov/#; The data processing and mapping of the map were completed using ENVI and ArcGIS.).

The land use pattern in the research area is also related to nitrate pollution in groundwater. From a spatial distribution perspective, high concentrations of nitrate are highly concentrated in the central area where cultivated land and construction land intersect, as well as in the distribution area of cultivated land in the northwest. In contrast, the southeastern region, which is mainly covered by vegetation, generally has lower nitrate concentrations, forming a sharp contrast between the two (Fig 9). The land use type is a direct reflection of human activities on the surface, and its spatial pattern directly controls the intensity, path, and distribution characteristics of nitrate inputs, providing a key basis for accurate identification of pollution sources.

Cultivated land, as the main land use type in the study area, is widely distributed in the flat areas along the Yellow River and is the core carrier of agricultural pollution. Long term intensive nitrogen fertilizer application and irrigation activities continuously migrate nitrogen that has not been absorbed by crops to groundwater through leaching, making it a common land use type for high nitrate groundwater [39]. The building land and cultivated land are intermingled in the central part of the study area, where point source pollution such as domestic sewage and manure discharge is combined with agricultural non-point source pollution, further exacerbating nitrate accumulation and forming a heavily polluted hotspot area which is also the most strongly disturbed by human activities [60]. The disturbance intensity of human activities in the vegetation covered area in the southeast is extremely low, and the nitrogen element in groundwater may come from atmospheric deposition, decomposition of organic matter in shallow soil, and leaching of nitrogen-containing rocks [61]. And the terrain in the area is undulating, which can effectively block the infiltration path of exogenous nitrate, so the concentration of nitrate in groundwater is relatively low. In addition, the nitrate concentration at points around the river is slightly lower than that of farmland far away from the river, indicating that river water recharge has a certain dilution effect on groundwater and plays a regulatory role in the pollution level along the Yellow River.

In summary, the factors affecting nitrate pollution in groundwater include both human and natural factors. Different rock types of aquifers have an impact on the enrichment and migration of nitrate, with loose rock pore water and carbonate rock fracture karst water being the water types severely polluted by nitrate. Farmland is widely distributed in the study area, and agricultural activities such as fertilization and irrigation can lead to a large amount of nitrate accumulation in groundwater. At the same time, the discharge of domestic sewage and fecal leakage in densely populated areas such as Xiaoli Town and Guide Town have become important reasons for the aggravation of local pollution. Therefore, the Xiaoli-Guide area in the southern part of the Yellow River has been identified as a potential source of nitrate pollution. The region is highly concentrated in agriculture and human activities, and the development of karst fissures leads to the convergence of nitrate migrated from upstream and nitrate input by local humans.

### 4.2 Regional comparison of nitrate contamination in the lower Yellow River Basin

The ecological protection and high-quality development of the Yellow River Basin is a major national development strategy in China. The lower reaches of the Yellow River Basin are important grain production centers and gathering areas of modern industry with dense population and active economy. NO_3_^-^ in groundwater threatens drinking water safety and public health, while also restricting the high-quality development of agriculture, thus identifying the current status of nitrate pollution in the groundwater in the lower Yellow River Basin is crucial for maintaining ecological balance and supporting socioeconomic development.

Zhang et al. [62] conducted a study on nitrate pollution in groundwater in Panzhuang Yellow River irrigation area in the western part of Dezhou City, Shandong Province. The results showed that long-term irrigation and fertilization activities led to excessive NO_3_^-^ content in groundwater. Approximately 10% of water samples exceeded the standard, and main reasons for exceeding the standard were feces and sewage, followed by chemical fertilizers, atmospheric precipitation and soil. Wei et al. [63] analyzed the nitrogen and oxygen isotopic composition of nitrate in Binzhou City by using nitrate nitrogen and oxygen double isotope and isotope mixing models. The results showed that the over standard rate of nitrate in groundwater in Binzhou City was 27.78%, and organic fertilizers and sewage were the main sources of nitrate pollution. This study, in conjunction with previous research, indicates that in the lower reaches of the Yellow River, agricultural activities, feces and sewage are the main causes of excessive nitrate levels in groundwater. To improve the groundwater quality in this area, it is necessary to reduce the excessive application of nitrogen fertilizer and improve the utilization rate of fertilizer. Meanwhile, it is necessary to improve the construction of sewage treatment facilities, strengthen the monitoring of groundwater quality in key agricultural areas and around towns to master the distribution and change trend of nitrate pollution.

### 4.3 Sensitivity analysis of health risks

Using Monte Carlo simulation, the impact of various parameters on the non-carcinogenic health risk assessment results (HQ) of nitrate in groundwater in the study area was clarified. A higher absolute sensitivity values indicates a greater influence of variables on risk assessment [53,64].

**Fig 10:**
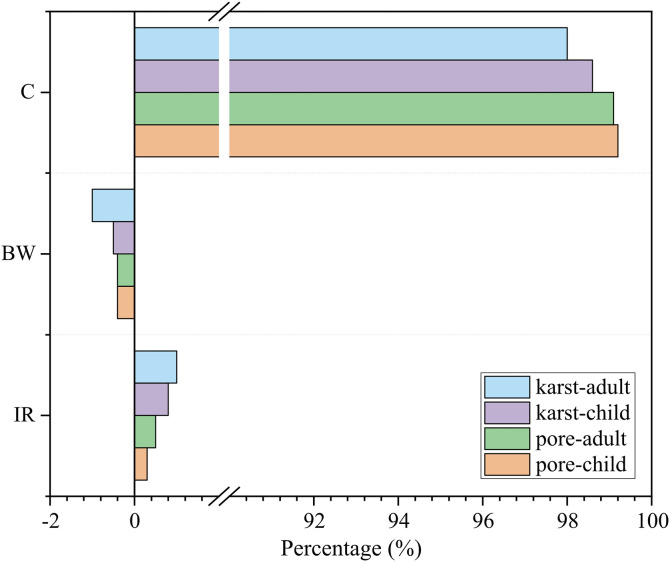
Differential impact of karst water and pore water on HQ sensitivity in adults and children.

From the perspective of overall sensitivity characteristics, nitrate concentration is a highly sensitive parameter that affects HQ results (Fig 10), and its impact on health risks is much higher than parameters such as IR and BW. The degree of nitrate pollution in groundwater is a key factor determining human health risks. From the perspective of differences in water body types, the sensitivity of nitrate concentration in pore water to HQ results is generally higher than that in karst water, which is related to the pollution characteristics of higher nitrate concentration and greater spatial variability in pore water. Small changes in its concentration will have a more significant impact on the health risk assessment results.

From the perspective of population differences, the sensitivity of nitrate concentration to children is significantly higher than that to adults. The effects of IR and BW on both adults and children are at a secondary level, and their overall contribution is relatively low. This pattern is closely related to the exposure characteristics of children and adults. Children have lighter body weight, consume more water per kilogram of body weight, and have a much higher risk of ingestion at the same nitrate concentration than adults. Therefore, their impact on children’s health risks is more prominent. At the same time, IR is positively correlated with HQ and BW is negatively correlated with HQ. Although their impact is relatively low, it also suggests that targeted exposure parameters need to be used when conducting health risk assessments for different populations to improve the accuracy of the assessment results.

In summary, the sensitivity analysis results indicate that the core of preventing and controlling the health risks of nitrate in groundwater in the study area lies in strictly controlling the concentration of nitrate in groundwater. At the same time, children should be identified as a key protected population, and targeted drinking water safety protection measures should be developed for children in high pollution areas to reduce the nitrate exposure risk of sensitive populations from the source.

### 4.4 Limitations and prospects of this study

Due to the limitation of sampling time only during the dry season, the nitrate pollution characteristics reflected in this study only represent the situation during the dry season. To more systematically characterize the chemical characteristics and pollution evolution of groundwater in the study area, future research should consider supplementing sampling during the wet season to obtain data under different hydrological conditions, thereby improving the overall understanding of regional water pollution. The exposure parameters used in the health risk assessment of this study mainly refer to previous research. In the future, data on the actual drinking habits and water use methods of the local population in the study area can be collected to establish a more regionally representative parameter distribution and reduce the uncertainty caused by parameters. At the same time, in addition to the conventional grouping of children and adults, sensitive populations such as infants, pregnant women, and the elderly can be further subdivided, and targeted exposure parameters can be used to identify high-risk populations.

## 5 Conclusions

The Changxiao hydrogeological unit is an important water supply source for Jinan City. Water quality safety is related to regional economic and social development and livelihood security. Nitrate is a key indicator affecting the quality of groundwater in this area. This study described the characteristics of nitrate pollution in groundwater and its health risk to human body through nitrate pollution index, multivariate statistical analysis and probabilistic health risk assessment. The main conclusions are as follows:

(1) The average concentration of NO_3_^-^-N in samples was 19.48 mg/L, with pore water samples showing the highest average nitrate concentration, at 26.61 mg/L, followed by karst water samples. Nitrate pollution was mainly distributed in densely populated areas, especially near Xiaoli Town, Guide Town and Ancheng Town.
(2) NPI of samples ranged from −0.992 to 14.15, with an average value of 3.31. 10.87% of the water samples were moderately polluted, while 50% of the water samples were seriously polluted by nitrate.
(3) The results of hierarchical cluster analysis and factor analysis show that the chemical characteristics of groundwater in the study area are affected by both natural and human factors. Under the water-rock interaction, a hydrochemical field with lithological characteristics has formed, while anthropogenic activities have significantly altered the original hydrochemical regime. Especially in areas with serious nitrate pollution and intensive anthropogenic activities, agricultural activities and the discharge of sewage and feces have caused a large number of nitrates to enter the groundwater.
(4) The probability health risk assessment showed that the probability of potential risk for children and adults in pore water was 24.37% and 6.67%, respectively. In karst water, the probability of potential risk for children and adults is 24.83% and 3.64% respectively. The potential risk of nitrate to adults is far less than that to children who should be paid more attention. At the same time, nitrate concentration was the main variable affecting the health risk assessment. In order to reduce the health risk to human, it is necessary to control the nitrate content in groundwater.

## Supporting information

S1 Data Data. (XLSX)
